# Microsatellite instability and mismatch repair protein deficiency: equal predictive markers?

**DOI:** 10.3389/pore.2024.1611719

**Published:** 2024-04-09

**Authors:** Maja L. Nádorvári, Gábor Lotz, Janina Kulka, András Kiss, József Tímár

**Affiliations:** Department of Pathology, Forensic and Insurance Medicine, Semmelweis University, Budapest, Hungary

**Keywords:** mismatch repair deficiency, microsatellite instability, immunohistochemistry, molecular testing, cancer

## Abstract

Current clinical guidelines recommend mismatch repair (MMR) protein immunohistochemistry (IHC) or molecular microsatellite instability (MSI) tests as predictive markers of immunotherapies. Most of the pathological guidelines consider MMR protein IHC as the gold standard test to identify cancers with MMR deficiency and recommend molecular MSI tests only in special circumstances or to screen for Lynch syndrome. However, there are data in the literature which suggest that the two test types may not be equal. For example, molecular epidemiology studies reported different rates of deficient MMR (dMMR) and MSI in various cancer types. Additionally, direct comparisons of the two tests revealed relatively frequent discrepancies between MMR IHC and MSI tests, especially in non-colorectal and non-endometrial cancers and in cases with unusual dMMR phenotypes. There are also scattered clinical data showing that the efficacy of immune checkpoint inhibitors is different if the patient selection was based on dMMR versus MSI status of the cancers. All these observations question the current dogma that dMMR phenotype and genetic MSI status are equal predictive markers of the immunotherapies.

## Introduction

MMR deficiency (dMMR), and the resultant microsatellite instability (MSI), is one of the most frequent genetic alterations of the DNA repair systems of cancer. It is most prevalent in the gastrointestinal (GI) tract (colorectal, gastric, and esophageal) and endometrial cancers but can occur at a much lower frequency in any other cancer types. Initially, they served only as a genetic marker for the hereditary cancer disease Lynch syndrome [1, 2], but now most tests for microsatellite instability are performed as predictive marker tests of immune checkpoint inhibitor (ICI) therapies [3–7]. Currently, the two main diagnostic methods of microsatellite instability are testing the expression of MMR proteins with immunohistochemistry (IHC) and molecular diagnostics of MSI mostly by polymerase chain reaction (PCR)-based techniques. Next-generation sequencing (NGS)-based detection has also become a tool to determine MSI status, as it can analyze more microsatellite markers and MMR gene mutations simultaneously, which can help in therapeutic decisions [8]. Immunohistochemistry can only reveal the absence of MMR proteins, which is excellent for Lynch syndrome. However, IHC cannot test the mismatch repair system from a functional aspect, which is a crucial factor in immune checkpoint inhibitor therapies, since this defect increases the mutational burden and the amount of neoantigens. In routine diagnostics, MMR IHC and MSI PCR are the most frequently used techniques, and their predictive values are equivalent according to the international guidelines. Molecular epidemiology data of dMMR and MSI demonstrated different incidences in various cancer types, suggesting that the two markers may not be equivalent. On the other hand, there are several smaller or larger studies that directly compared various testing methods of dMMR and MSI and indicated smaller or larger differences in sensitivities, specificities, and predictive values. Since the breakthrough investigation of immunotherapies, the diagnostic aim of MSI/MMR status have overgrown Lynch syndrome. It is important to consider that most diagnostic tools on the market now are optimized for colorectal carcinomas (CRCs), so they are not necessarily equally sensitive in other cancer types. The goal of this review is to analyze the prevalence of MSI-high/dMMR status in various cancer types, the current detection strategies, and their discrepancies and validity in the light of prognostic value for immunotherapies.

## Mismatch repair deficiency and resultant microsatellite instability

DNA repair deficiency is a hallmark of cancer and may involve all the possible forms, namely homologous recombination repair (HRR), mismatch repair (MMR), nucleotide excision repair (NEJR), and base excision repair (BER), but current clinical attention is on MMR and HR deficiencies [9]. There are ∼2 × 10^7^ microsatellites present in the whole genome, including exomes (∼5 × 10^5^), the non-coding regions, and the introns which make up 3% of the genome [10, 11]. Every microsatellite consists of repetitive sequences in which the aberrant shift in the length of the microsatellites usually occur during replication [12]. Microsatellites are highly conservative parts of the genome, and they are exceptionally prone to mismatched base pairing and frameshift mutation. Accumulation of them leads to increased tumor mutational burden (TMB), an increased neoantigen load and, as a consequence, stronger antitumoral immune responses [13]. All these consequences of microsatellite instability make it an excellent predictive biomarker for immune checkpoint inhibitor therapies [14]. It is of note that cancers share the most frequently involved microsatellite markers, but they are also characterized by their own unique sets involved in MSI [11] (Table 1).

**TABLE 1:** Most frequently involved microsatellite markers in various cancer types [11].

COAD	READ	STAD	UCEC
ACVR2A	ACVR2A	ACVR2A	
ATPEV0A2	ATPEV0A2	ATPEV0A2	
DEFB105A/B		DEFB105A/B	DEFB105A/B
NOMO1		NOMO1	
AP4S1		AP4S1	
TIAL1		TIAL1	
UBR5		UBR5	
TM2D1		TM2D1	
BRAF	BRAF	PIP5K1A	PIP5K1A
		TAF2	TAF2
		PDE4DIP	PDE4DIP
CRP2	AHCYL2	MACF1	
BMPR2	PLCB4	DOCK10	
	IQGAP1	KNOP1	
	SCLT1	GTF2H2B	
	EFCAB5	NMS	
	ARHGEF15	UTP20	
	ABLIM1	HMCN1	
	APH1B	NLN	
		CACNA1D	

COAD, colon adenocarcinoma; READ, rectal adenocarcinoma; STAD, stomach adenocarcinoma; UCEC, uterine endometrial adenocarcinoma (bold: cancer-specific patterns).

The MMR system corrects spontaneously arising somatic mutations and is indispensable for the reduction of mutational rate. Its impaired function results in the loss of the integrity of the genome and promotes malignant transformation, which is even more accentuated in the germline mutations of the MMR system. The main MMR proteins are MLH1, MSH2, MSH6, and PMS2, but MSH3, MLH3, and PMS1 can also be important in special circumstances [9, 15]. The MMR proteins repair the incorrectly paired bases, thus the ones which did not form cytosine-guanine or thymidine-adenine pairs. These mismatches are mostly caused by replicational errors but also by oxidative stress, lipid peroxidation, or methylation and alkylation [16]. The MMR proteins function as heterodimers: MLH1 and PMS2 form MutLalpha while MSH2 and MSH6 form MutSalpha. The activation of MutSalpha complex is dependent on ATPase activity, which is crucial for the connection with the mismatched DNA and the initiation of the reparation. A flawed DNA string is bound to MSH6 by the mismatched base pair as a part of MutSalpha. There is an alternative complex, MutSbeta, consisting of MSH2 and MSH3 in a 1:10 ratio to MutSalpha and it usually binds larger defects [16, 17]. The binding of MutSalpha stimulates ATP hydrolysis and facilitates the binding of MutLalpha, thus the tetramer of the four mismatch-correcting protein can evolve. The MMR pathway is bidirectional. The 5′-end binds the newly synthetized strand, allowing exonuclease 1 (EXO1) and ATP binding with MutSalpha and MutLalpha to carry out conformational changes on DNA. The MutSalpha/MutLaplha/EXO1 complex facilitates the function of RPA (replication protein) and PCNA (proliferating cell nuclear antigen) so these enzymes can repair the mutations. 3′-5′ repair also exists, but our knowledge on it is limited. Alternatively, MLH1 can form dimer with MLH3 (resulting in MutLγ complex), which has a role in meiotic recombination [17]. MLH1 can also form another alternative dimer, MLH1/PMS1, resulting in the MutLβ complex. The MMR system arrests cell cycle when it detects any defect. It is possible that, after revealing the error in the freshly synthetized strand, the mismatch repair system tries to cut them out and, if it fails, the original strand will break as well, inducing the ATM/ATR/p53 cell death pathway. Alternatively, it is possible that MutSalpha and MutLalpha signal the cell death pathway directly after the detection of mismatches [18, 19]. The mutation rate of MMR genes is different by cancer types and include not only the common MMR genes (MLH1, MSH2, MSH6, and PMS2) but also the minor ones (MSH3, MLH3, and PMS1). Analysis of the TCGA database indicates that one-third of MMR gene mutations affect these minor MMR genes. MMR gene mutation frequency is the highest in endometrial adenocarcinoma (>50%), followed by gastric (>30%), colorectal, and ovarian cancers (>20%) (Table 2).

**TABLE 2:** Incidence of MMR gene mutations in various cancer types in TCGA database.

MMR gene	Endometrial adenocarcinoma	Gastric adenocarcinoma	Colon adenocarcinoma	Rectal adenocarcinoma	Ovarian carcinoma
MLH1	7	3	4	1	2
MSH2	9	3	4	2	3
MSH6	12	3	5	2	3
PMS2	8	5	3	1	3
mutation rate	36	14	16	6	11
MSH3	10	4	2	3	5
MLH3	2	4	4	0	1
PMS1	6	3	3	1	5
total MMR mutation rate	54	35	25	10	22

Data are expressed in %. MMR, mismatch repair.

## MMR deficiency during tumor progression

A large cohort of 100,000 colorectal cancer patients in the US was analyzed for MSI/dMMR prevalence according to stages and demonstrated a gradual decrease of incidence from stage I to stage IV [20]. A large meta-analysis of MSI/dMMR literature also indicated that the MSI prevalence in colorectal cancers decreased significantly from Stage I/II to Stage III/IV in Japan and South Korea while no such changes were reported in endometrial or ovarian cancers [21]. Contrary to what would be expected if we consider MMR deficiency as a founder genetic alteration during CRC progression, data may suggest that, during tumor progression, MMR deficiency is lost in a significant number of cases. To test this directly, the MSI/dMMR genetic status was compared in a ∼100 patient cohort in primary tumor and their metastases. This analysis found the loss of MSI/dMMR in CRC metastases was a rare event (3.4%) [22]. An analysis of a small number of MSI-high cancers (six different types) directly evaluated the clonal development during tumor progression by using multiregional tumor sequencing. This analysis did not find loss of MSI clones in various types of metastases but rather detected progressive changes in the forms of MSI paralleled with an increase in TMB and the emergence of new driver mutations in various clones. These data seem to be contradictory unless the biology of MSI/dMMR colorectal cancers is different [23]. A study of a large cohort of CRC patients analyzed the survival after recurrence (SAR) of >2,500 patients and found that MSI/dMMR patients have a significantly longer survival compared to MSS/pMMR ones [24]. Biologically, MSI/dMMR colorectal cancers tend to be less metastatic, although they are resistant to 5-FU-based chemotherapies [15].

## Diagnostic techniques of dMMR/MSI

### Immunohistochemical diagnosis of MMR deficiency

Loss of expression of mismatch repair proteins can be detected by immunohistochemistry. The currently recommended method applies detection of four MMR proteins, which is an excellent tool for the original aim of MMR testing: detecting Lynch syndrome [17, 25]. These proteins work in heterodimers, so in the majority of dMMR cases, double loss of MLH1 and PMS2 or MSH2 and MSH6 is present, which is called the classical dMMR [26]. When MLH1 or MSH2 is mutated or degraded, it will cause the loss of PMS2 or MSH6 as well, thus the isolated loss is more frequent in the last two proteins [27, 28]. This is due to the compensatory function of other MMR proteins such as MSH3 for MLH1 and MSH2 or PMS1 for MLH3. MLH1 promoter hypermethylation can also cause dMMR phenotype [15]. Immunohistochemistry of the MMR proteins is performed on FFPE blocks by using the CE-marked kit Ventana-MMR-RxDx^1^ or Dako/Agilent-set [29] containing antibodies to MLH1, MSH2, PMS2, and MSH6. Two types of positive controls have to be used: a positive control sample is necessary while tumor stromal fibroblasts and lymphoid cells serve as internal controls. These latter cells are extremely important since their positivity indicates that the sample is correctly preserved while weak or lost nuclear staining of normal cells indicates severe preanalytical problems such as over- or underfixation or long hypoxia times that will lead to equivocal interpretation [30–33] (Table 3).

**TABLE 3:** Four-protein MMR IHC evaluation protocol [www.nice.org.uk/guidance/dg42, [34]].

Inner control nuclear	Positive	Positive	Positive	Positive	Negative	Negative
tumor nuclear	+>90%	−>90%	Unusual >10%	T+ weaker than IC+	negative	Positive any type
variants	−	A/B/C	A/B/C	A/B/C		
interpretation	pMMR	dMMR	dMMR	equivocal	equivocal	equivocal

A: classical ML1/PMS2 or MSH2/MSH6 double loss, B: single MMR protein loss, C: multiple (>2) MMR protein losses, IC, inner control cells; dMMR, mismatch repair deficient; pMMR, mismatch repair proficient; T, tumor cells; unusual, clonal loss; heterogeneity etc.

**FIGURE 1:**
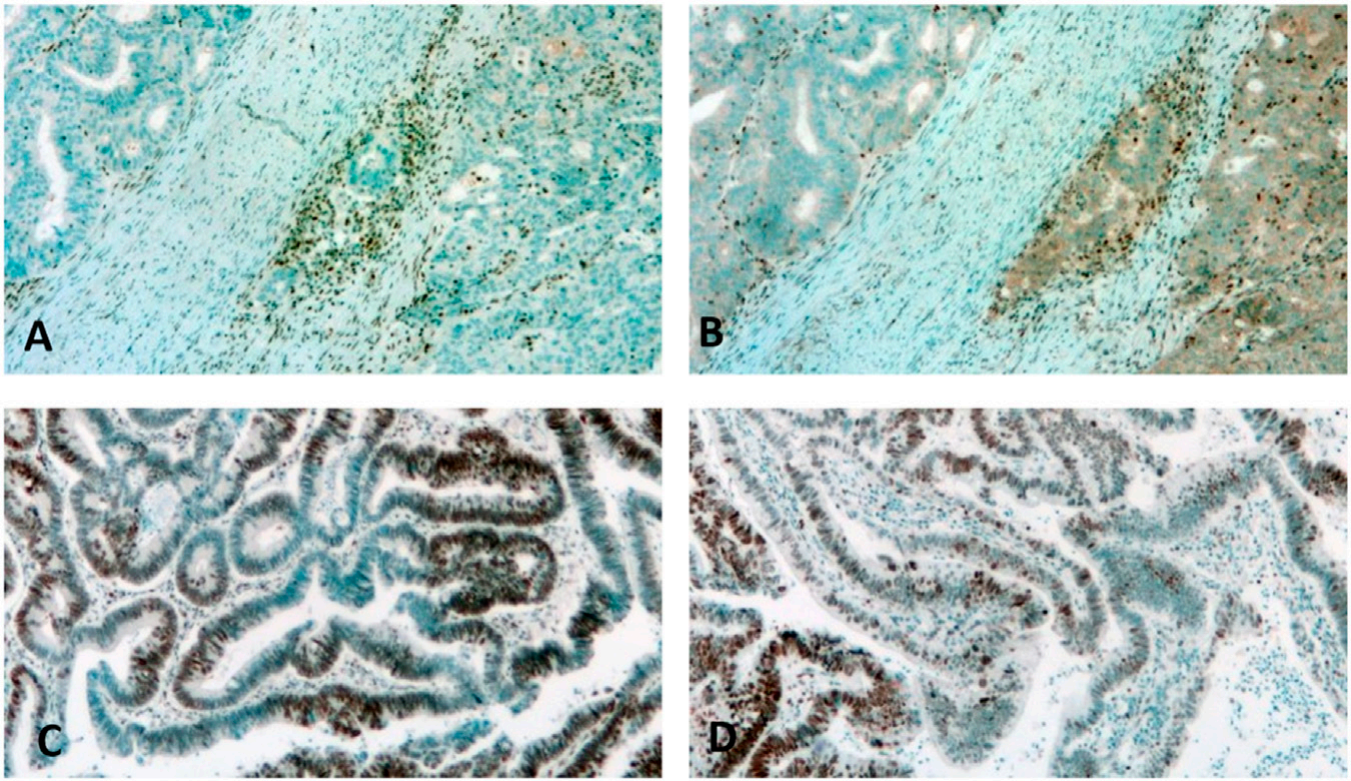
Detection of dMMR by immunohistochemistry. **(A,B)**: Classic two-protein loss in colon cancer. Note the intense labeling of stromal cells. **(A)**: MLH1, **(B)**: PMS2. **(C)**: Focal loss of MSH2 in a colon cancer case. Note the intense staining of stromal normal cells adjacent to the negative tumor cells. **(D)**: Heterogenous loss of MSH6 in a rectal cancer case. Note the intense staining of the stromal cells adjacent to the negative tumor cells.

dMMR can be diagnosed as classical two-protein losses (MLH1/PMS2, Figures 1A, B, or MSH2/MSH6 losses), non-classical single or multiple losses in >90% of tumor cells, or as unusual (focal/subclonal or heterogenous losses of >10% of tumor cells in the background of positive stromal cells [10, 15, 34–36] (Table 3; Figures 1C, D). In some previous reports, abnormal IHC staining was divided into three intensity categories: strong, intermediate/equivocal/unusual, and complete loss/negativity. An indeterminate IHC phenotype was found to occur at a low rate of ∼3–6% [2, 37, 38], but in one study the incidence was reported to occur at 15% [26]. In case of negativity of the normal stromal cells, the test must be repeated and, if still negative, the case must be considered equivocal, irrespective of the tumor cell nuclear staining [30, 34] (Table 3). It seems that the most challenging situation is when there are differences in the staining intensities of control stromal cells and tumor cells, since in those cases the diagnosis is equivocal but can easily be considered unusual. Although the MMR IHC is considered to be a four-protein detection, in certain analyses five- or six-MMR-protein detections were performed by IHC using anti-MSH3 and anti-MLH3 antibodies [39]. On the other hand, there are several reports which follow a two-antibody staining protocol of using MSH6 and PMS2 as an initial method and determinations of MSH2 and MLH1 are performed only in cases of loss or abnormality [40].

### Sequencing of MMR genes

Loss-of-function mutations of MMR genes can be detected by classical Sanger sequencing but is done more frequently by NGS using focus panels which contain all six MMR genes as well as BRAF and EpCAM. The advantage of NGS is that it can detect loss of heterozygosity (LOH) of an MMR gene as well. It can be performed using tumor tissue, but in cases of mutations with high variant allele frequency, germline testing must be done to identify Lynch syndrome patients. However, in cases of mutations of VUS (variant of uncertain significance) category, further analysis of the functional consequence must be carried out to detect microsatellite instability.

### Methylation analysis of MMR gene

In cases of MLH1/PMS2 protein losses in tested cancers, beside the loss-of-function mutation of MLH1, promoter methylation may also be the genetic cause; therefore, a methylation analysis can be performed with in-house PCR protocols or using commercial kits such as SALSA MS-MLPA [41].

### Diagnosis of microsatellite instability (MSI)

#### PCR-based techniques

The gold-standard test for microsatellite instability is fluorescent polymerase chain reaction which detects the instability (variability of length) of the tested microsatellite markers with a technical sensitivity of ∼10%. The most frequently used MSI-PCR kits apply five microsatellite markers (Promega Pentaplex). One is the Bethesda panel (Table 4) with three dinucleotides and two mononucleotides designed to test Lynch syndrome [2, 17, 43]. The improved version of this Pentaplex panel uses five mononucleotide markers (Table 4). The tumor cell/normal cell (T/N) ratio is critical for assay performance, since 20% can be used as a cut-off. In case of low T/N ratio, a microdissection can be used to improve it. DNA is isolated from tumor tissue and from normal tissue; an MSS cell line can also be used. After PCR reaction, the fragment length analysis can be performed. According to international recommendations, samples are defined as MSI-high if two or more markers are unstable (≥40%); MSI-indeterminate (MSI-low) is diagnosed if one marker is unstable (20%) [2, 15, 34].

**TABLE 4:** Microsatellite markers used in MSI diagnostics.

TCGA [11]	TCGA [42]	Bethesda [43]	Pentaplex [43]	Idylla [41]	Easy PGX [44]	MSI-Titano [45]	NGS-10 [46]
DEFB105A/B	ACVR2A	BAT25 (KIT)	BAT25 (KIT)	ACVR2	BAT25	BAT25	KDM6A
ACVR2A	TGFBR2	BAT26 (MSH2)	BAT26 (MSH2)	BTBD7	BAT26	BAT26	SMARCB1
RNF43	RNF43	D5S346	NR21 (SLC7A8)	DIDO1	NR21	BAT40	GRIN2A
DOCK3	RPL22	D2S123	NR24	MRE11	NR22	NR21	FLT1
GTF2IP1	MLL3	D17S250	MONO27 (BIRC3)	RYR3	NR24	NR24	CDK4
ARHGEF12	PRDM2			SEC31A	NR27	D2S123	KTM2A
NOMO1	JAK1			SULF2	MONO27	D17S250	KIF5B
PIP5K1A	APC				CAT25	D5S346	BCL2L11
KIF14						D18S58	MSH6
DDX59						TGFBR2	EML4
		MSI-H cut-off	MSI-H cut-off	MSI-H cut-off	MSI-H cut-off	MSI-H cut-off	MSI-H cut-off
top MS markers	top MS markers	2 (40%)	2 (40%)	2 (28.6%)	2 (25%)	4 (40%)	3 (30%)

MS, microsatellite; MSI-H, microsatellite instability-high.

Idylla™ MSI is another PCR test, which is an automated analysis system. It also works from FFPE blocks and there is no need for control tissue. The Idylla™ system uses seven microsatellite loci (Table 4). At least five markers must be detectable to be able to evaluate the case and MSI-H is defined if a minimum of two unstable markers (28.6%) are detected [41, 47]. Another assay uses eight microsatellite markers, again a combination of the two Pentaplex systems (EasyPGX) [44]. Another automatic PCR test system is the Titano MSI, which uses 10 microsatellite markers, a kind of combination of the Bethesda and Pentaplex marker systems with similar evaluation criteria for MSI-H (≥40% [45] (Table 4). Last but not least, NGS-based MSI tests have also been developed using the Pentaplex markers or 10 different microsatellite markers (Table 4) and various MSI-H diagnostic algorithms, mostly using 30% as an MSI-H cut-off value [46].

#### Large gene panel-based MSI determinations

There are several FDA-approved CAP-accredited large gene panel-based NGS tests available to assess MSI. The F1Dx assay is based on 1,880 mononucleotide homopolymers and allelic length variability can be computed, with the assessment performed by principal component analysis using a 30% cut-off value for MSI [48]. The MSK-Impact assay applies 1,581 MS loci to assess variability in length and the sequencing data are analyzed by MSI sensor where the % of unstable loci is provided as a score, with the cut-off for MSI at 20% [37, 49]. The Illumina TruSight Oncology 500 system evaluates 1.24 Mb genome and is based on 130 MS marker sites evaluated by Dragon-MSI, where the cut-off is set at 20% [50]. A similar NGS-based MSI test is also available from Thermo Fisher Scientific: Oncomine Comprehensive Assay Plus-MSI, where 76 microsatellite markers can be tested, including the Bethesda and Pentaplex ones. Sequencing data are analyzed by MSICall software. In this system, a tumor-type specific score was applied with cut-off ranges of 19.7%–32.9% [51]. Furthermore, MSI analyses based on whole genome sequencing (WGS) or whole exome sequencing (WES) are also possible using many different bioinformatic tools, extensively reviewed in [32].

## dMMR/MSI as predictive markers for immune checkpoint inhibitors and the Lynch syndrome: recommendations

According to the recommendations of ESMO for MMR/MSI testing as a predictive marker for immunotherapy, IHC is the first test of choice, using four antibodies for MMR proteins (MLH1, PMS2, MSH2, and MSH6). It is important to keep in mind that these proteins form heterodimers and the most trustworthy pattern is when the protein expression is lost in pairs of MLH1/PMS2 or MSH2/MSH6 (called the classical pattern). ESMO recommends PCR testing in case of any doubt in IHC or confirmatory analysis. The Bethesda panel of two mononucleotides (BAT-25 and BAT-26) and three dinucleotides (D5S346, D2S123, and D17S250) and the Pentaplex panel of five mononucleotides (BAT-25, BAT-26, NR-21, NR-24, and MONO-27) can be used to determine MSI status. However, the Pentaplex panel is recommended since it is characterized by higher sensitivity and specificity. In cases of colorectal cancer, IHC and PCR tests are both recommended but for non-Lynch syndrome cancers data are limited for reliability; therefore, NGS-based techniques are recommended [52].

The College of American Pathologists (CAP) separates testing methods by cancer types. In colorectal carcinoma, IHC and PCR are considered equivalent for MSI/dMMR testing. NGS can also be used. For gastroesophageal and small bowel cancers, pathologists can use MSI PCR, MMR IHC, or NGS for diagnosis. In cases of endometrial cancer, MMR IHC test is the first choice. For cancer types other than those mentioned above, MMR proteins should be tested, but the optimal approach has not yet been established. TMB should not be used as a surrogate for DNA MMR defects; MMR IHC or MSI PCR must be performed to determine whether the TMB-high status is secondary to MMR deficiency [34]. The recent ASCO guideline for immunotherapy diagnostics suggested MMR-IHC, MSI-PCR, and NGS for colorectal cancer, MMR-IHC and MSI-PCR for other GI tract cancers, and only MMR-IHC for endometrial cancer. In case of any other type of non-GI tract cancers, no particular test method was recommended when testing for MMR deficiency/MSI [53].

German pathologists also created a guideline for MMR/MSI testing. They emphasized dMMR phenotypes and the importance of their interpretation. Their suggestion is that complete expression of all four proteins is normal, dMMR is the classical dual loss of MMR proteins MLH1/PMS2 and MSH2/MSH6, any other type of MMR losses should be considered abnormal, and it is advised to perform alternative diagnostic tests. If in doubt, it is important to check all preanalytical factors and the test should be repeated or the protocol be adjusted. According to them, MMR protein IHC outperforms MSI-PCR for non-CRC tumors. Supplementary molecular testing should be performed for further clarification of MMR-IHC if it is ambiguous or unusual. MLH1 methylation and mutation testing and NGS should be performed to determine MMR gene mutation status. NGS can be used only if it is validated against PCR or IHC. IHC is superior to PCR in non-colorectal cancer samples because PCR tests are mostly optimized for CRC [54].

The Colorectal Pathway Group (UK) also created a diagnostic approach for MSI/dMMR colorectal cancers. They divided CRC cases into two groups. Group 1 is all colorectal cancer patients aged under 50 years old. Group 2 A) consists of cancers with histologic features suspicious of Lynch syndrome and patients with family history of CRC, group 2 B) is cases with an oncologist’s request. The pathway for MMR testing starts with any group 1 tumors or requested referral from a molecular team, where a consultant surgeon or oncologist should be responsible for the patient and a clear referral must be made. A tumor block must be provided, preferably in continuity with normal tissue as well as a separate block of normal tissue, along with sufficient demographic data and the copy of the original histopathologic report. Tumor materials are assessed for loss of MLH1, PMS2, MSH2, and MSH6 proteins. Samples demonstrating MLH1 with or without PMS2 loss by IHC must be sent for genetic analysis for MLH1 promoter methylation, MLH1, and BRAF mutation statuses. The report of MMR-IHC or MSI-PCR must be a clear statement and will be sent for referral to a histopathologic department. Patients with loss of MSH2 and/or MSH6 or with loss of PMS2 alone are at high-risk of Lynch syndrome and should be referred and tested for the appropriate MMR gene mutation^2^.

## Molecular epidemiology of dMMR and MSI: discrepancies

The incidence of MSI in human cancers can be defined best on the basis of data available from the TCGA where 2 × 10^7^ microsatellite markers can be analyzed genomically. There are several major analyses performed using different bioinformatic tools to define MSI frequency. The common theme of these studies is that all of them used MSI-H defined by multiplex PCR as a reference (Bethesda and/or Pentaplex [11, 42, 48, 55–57]. All these studies found that endometrial cancer has the highest incidence of MSI (three studies: 28.3%–31.4%, one at a significantly higher rate) (Table 5). Gastric cancer is in the second highest (MSI 18%–20%), closely followed by colon cancer (16.6%–19%). In fourth place we find rectal cancer, where the incidence rates are spread from 3% to 9.2%. A large study corrected these figures to 2.7% based on dMMR [58]. In fifth place of the MSI list is ovarian cancer, with a lower frequency of 2%–5%. It is of note that adrenocortical, esophageal, liver, and cervical cancers are also found to have a relatively high incidence of MSI in TCGA (3%–5%). It is equally important that in melanoma, thyroid, and pancreatic cancers MSI is extremely rare [11, 42]. An analysis of a very large patient cohort using the FDA-approved F1Dx MSI test found significantly lower incidences of MSI in cancers of the highest frequencies, where the reference was MSI-PCR or MMR protein IHC [48] (Table 5). Meanwhile, if we compare the MSI frequency in various cancer types to the incidence of the MMR mutations we can see that the mutation rate of MMR genes in endometrial, gastric, colorectal, or ovarian cancers is significantly higher than the incidence of MSI determined by NGS, which is interesting since MMR genes can be turned off not only by mutations but also by methylation, which would result in a higher MSI rate than MMR gene mutation (Table 5). This discrepancy deserves further attention, since it seems that MMR gene mutation does not automatically lead to MSI at genome level.

**TABLE 5:** Molecular epidemiology of MSI as determined by NGS in TCGA using various bioinformatic tools.

Cancer	Mozaik [11]	Sputnik [42]	Mantis [55]	mSINGS [56]	F1Dx [48]	MMR mutation rate
UCEC	29.5	28.3	31.4	40	16.5	54
COAD	18.5	16.6	19.7	19	4.4	25
STAD	18	21.9	19.1	20	3.4	35
READ	3	9.2	5.7	5		10
OVCAR	2	3.2	1.4	5	0.7	22
reference	multiplex PCR	multiplex PCR	multiplex PCR	multiplex PCR	dMMR/MSI-H	

Data are expressed in % of MSI tumors. MMR genes: MLH1, MSH2, MSH6, PMS2, MSH3, MLH3, and PMS1.

dMMR, mismatch repair deficient; MSI-H, microsatellite instability-high; COAD, colon adenocarcinoma; OVCAR, ovarian carcinoma; STAD, stomach adenocarcinoma; READ, rectal adenocarcinoma; UCEC, uterine endometrial carcinoma.

Two major meta-analyses have been performed on the frequency of MSI (and dMMR) in various cancer types [21,59], Which indicate that, as compared to TCGA analyses, the incidence of MSI-H (as defined by PCR) in the top cancer types are under-reported by a factor of 30%–50%. It is of note that in these analyses the dMMR incidences (detected by IHC) frequently differ from MSI by a factor of 16%–40%. Another meta-analysis of the incidence of MSI/dMMR was performed in ovarian cancer, where the MSI incidence is low in TCGA [60]. This analysis found that the MSI incidence (determined by PCR) was in the range of that in TCGA (6%) but, disturbingly, the incidence of dMMR was twice as high as that of MSI [60]. These data are suggestive but are not based on the direct comparison of PCR and IHC tests on the same patient population and on the same cancer type.

## Direct comparison of various diagnostic techniques

Diagnostics of Lynch syndrome was developed well before the widespread use of MMR/MSI marker testing for therapeutic purposes. It was based on MMR immunohistochemistry followed by functional tests for MSI and, ultimately, sequencing germline MMR genes. This led to the generally accepted perception of MMR IHC as the gold standard of dMMR testing, supported by various pathological guidelines in which molecular techniques for MSI are recommended mainly in so-called equivocal cases. Meanwhile, MSI incidence in various cancers was defined by WGS or WES technologies by analyzing TCGA or other databases where the reference tests were Bethesda or Pentaplex PCRs [11, 42, 48, 55, 56]. Various analyses suggested a 5%–10% error rate of MMR protein IHC and MSI PCR, resulting potentially in 10%–20% discordance rates [13, 32]. Earlier studies demonstrated that a relatively high proportion (∼30%) of equivocal dMMR cases are MSI-high by PCR and have Lynch syndrome [2], while 6% of MSI-H cancers retain the expression of MMR proteins studied by IHC [37], suggesting a more complex relation between dMMR and MSI. A large study of colorectal cancers identified a very low discrepancy rate (1.6%) of MMR IHC and MSI PCR where both the MMR test and MSI PCR were not homogenous; it used non-standard approaches and was corrected post-diagnosis [61] (Table 6). In a smaller colorectal cancer cohort, four-protein MMR IHC and Pentaplex MSI PCR results were compared, with a similarly low discrepancy rate (1.2%) [62] (Table 6). A comparative analysis of the four-protein MMR IHC to Bethesda MSI PCR in a large colorectal cancer cohort, however, found low correlation (∼0.5) and low sensitivity (81.1%) due to low correlation of MSI-H with dMMR [63]. A recent analysis of the four-protein IHC and the Pentaplex PCR tests, performed on a large series of colorectal cancers, found a high discrepancy rate of ∼18%, mostly in case of the non-classical and especially of the unusual immunophenotypes [64] (Table 6).

**TABLE 6:** Comparison of various MSI test types to MMR IHC.

Comparison	N of patients	Discordance (%)	Sensitivity (%)	Specificity (%)	Reference
colorectal cancer					
PCR to IHC4	3,228	1.6	NA	NA	61
Pentaplex to IHC4	593	1.2	NA	NA	62
Bethesda to IHC4	569	8.1	81.1	92.7	63
Pentaplex to IHC4	543	18.0	41.4	98.5	64
PCR8	28	NA	100	100	42
Idylla		NA	100	100	
NGS to IHC4		NA	100	100	
gastric cancer					
PCR7 to IHC4	488	0.8	100	92.9	65
endometrial cancer					
PCR to IHC4	696	4.6	NA	NA	66
PCR8	21	NA	58	100	42
Idylla		NA	67	100	
NGS to IHC4		NA	75	100	
Idylla to IHC4	100	13.3	80.4	100	47
pan-cancer non-colorectal					
Bethesda	185	NA	77.3	94.5	46
Idylla to IHC4		NA	68.2	91.4	
Pentaplex to IHC4	160	23.8	32.0	96.4	64

IHC4, four-protein immunohistochemistry; PCR7, seven MS marker PCR; PCR8, eight MS marker PCR.

A large study on gastric cancer reported a very low discrepancy rate of four-protein MMR IHC with Pentaplex PCR [65].

Analysis of a large cohort of endometrial cancers by MMR IHC and a variant of MSI PCR (a combination of Bethesda and Pentaplex panel) found 4.6% discordance, which was higher in MSS-MSI-L cases but was very low for MSI-H [66]. Other studies on endometrial cancer compared various MSI test types to IHC. Interestingly, the sensitivity of PCR, Idylla, or NGS tests was relatively low (58%–75%) [41, 46] (Table 6).

A large NGS-based analysis of tumors associated with Lynch syndrome found that non-colorectal/non-endometrial cancers were characterized in ∼30% by MSI-low status (determined by NGS) and were all dMMR by IHC [67]. In other study on non-colorectal cancers, the Pentaplex PCR exhibited a low sensitivity but high specificity using IHC as gold standard [64].

The comparison of Bethesda PCR and Idylla tests to IHC in another pan-cancer non-colorectal cohort found the MSI tests to have high specificities but much lower sensitivities [45] (Table 6).

All these data point to the fact that MMR IHC and MSI PCR-based techniques are not as equal as once thought. Even a relatively high concordance rate is not necessarily sufficient in daily routine diagnostics if the sensitivity or specificity are not high. Potential factors affecting these discordances are technical and preclinical factors (mostly hypoxia time and fixation issues) [31] and the low T/N cell ratios of the tumor sample [32], but it is now evident that there must be other, yet unidentified genetic/biological factors as well behind those differences. One of those factors could well be the genetic differences between Lynch-associated cancers and non-Lynch cancers.

## Clinical observations

Immune checkpoint inhibitor trials followed a three-way protocol by design according to predictive biomarkers in case of colorectal cancer. Usually in phase II trials patient selection was based on MSI-H (pembrolizumab: NCT01876511, nivolumab + ipilimumab: NCT03026140, durvalumab: NCT02227667, avelumab: NCT03186326). In the majority of the phase III trials patient selection was extended to dMMR/MSI-H (pembrolizumab: NCT02563002, nivolumab + ipilimumab: NCT04008030) or restricted to dMMR (atezolizumab: NCT02997228). In some trials, MSI-H-based selection was completed with TMB (BAT1306:NCT03638297) or POLE mutation (durvalumab: NCT13435107). However, the registration trials of pembrolizumab and nivolumab in colorectal cancer paved the way for dMMR/MSI-H biomarker pairs, which was followed by the tumor-agnostic indications [3–8]. None of these later trials have separately compared the dMMR and MSI-H biomarkers for therapy efficacy. Later analysis of trial data revealed that dMMR patients can be divided by MSI levels into a broad spectrum (MSIsensor NGS-based MSI analysis) and higher MSI levels were associated with higher response rates while progressing disease patients had significantly lower MSI levels in CRC patients (n = 36) [68]. In a different GI-tract cancer cohort (*n* = 6) of ICI-treated dMMR patients, survival upon treatment was significantly associated with the level of MSI [68]. In another small cohort of colorectal cancer patients (*n* = 33) treated with anti-PD-1 antibodies, treatment efficacy (ORR and PFS) was assessed according to the level of MSI determined by the Bethesda panel (patient selection was based on either dMMR or MSI-H). In this analysis no response was detected in MSS and MSI-L tumors and a significantly longer PFS was observed in patients with tumors of MSI≥3/5 marker positivity. It is of note that the three dMMR/MSS cases showed no response to immunotherapy [69]. Actually, this is the only study that suggested that MSI PCR could be a better predictor than MMR IHC.

A retrospective analysis of metastatic colorectal patients treated with immune checkpoint inhibitors evaluated dMMR and MSI-H (as determined by NGS) for efficacy. In this analysis both dMMR and MSI-H were positive predictors of PFS and OS in addition to TMB-high status. However, when MSI was added to dMMR, the predictive power increased for PFS and OS but dMMR addition to MSI did not influence the predictive power of MSI [70]. In a similar analysis on endometrial cancers, statistical evaluation of the superiority of MSI (as determined by NGS) vs. MMR for immune checkpoint inhibitor efficacy (time to treatment discontinuation, time to next treatment, and OS) was observed, whereas a superiority analysis for MMR vs. MSI was negative [71]. In this study, the concordance of IHC with NGS was found to be 91%, however, in the discordant cases NGS results better predicted therapy efficacy than IHC results.

There are data to support the value of MSI level as a predictor of immune checkpoint inhibitor therapy efficacy. A large cohort of MSI-H colorectal patients treated with ICIs was tested for TMB and a possible association with therapy efficacy. In this large analysis, it was found that MSI-H tumors of high TMB level (>37/Mb) were exclusively responders with longer PFS than the non-responder low-TMB patients [72]. A similar association of better survival and MSI-H and high TMB (>10 m/Mb) was observed in a large cohort of gastroesophageal cancer patients treated with immune checkpoint inhibitors [73]. According to these data, dMMR tumors can be subclassified by the level of MSI. Secondly, publications so far suggest that MSI is a stronger predictor of ICI therapy efficacy than dMMR.

## Conclusion

MMR deficiency is one form of the oncogenic alterations of the DNA repair systems. MMR deficiency is the cause of the inheritable Lynch syndrome, which leads to accelerated development of colorectal, endometrial, and other cancer types. On the other hand, MMR deficiency is used nowadays to identify patients eligible for immunotherapies due to the tumor agnostic indications of several immune checkpoint inhibitors. Current clinical guidelines recommend MMR protein IHC or the molecular MSI tests as predictive markers of immunotherapies. Most of the pathological guidelines consider MMR protein IHC as the gold standard test to identify cancers with MMR deficiency and recommend molecular MSI tests only in special circumstances and to screen for Lynch syndrome. However, there are data in the literature which suggest that the two test types may not be equal. Molecular epidemiology studies reported different rates of dMMR and MSI in various cancer types. In addition, direct comparisons of the two tests revealed relatively frequent discrepancies between MMR IHC and MSI tests, especially in non-colorectal and non-endometrial cancers and in cases with the unusual dMMR phenotype. Further, there are scattered clinical data showing that the clinical efficacy of immune checkpoint inhibitors is different if the patient selection was based on dMMR versus MSI status of the cancers. All these observations question the current dogma that dMMR phenotype and genetic MSI status are equal predictive markers of immunotherapies. It is time to consider international efforts to answer these questions and correct clinical and/or pathological guidelines if necessary.

## Footnotes

1
www.diagnostics.roche.com

2
https//mft.nhs.uk.app.uploads2018/09
